# Shedding Kinetics of Infectious Hematopoietic Necrosis Virus (IHNV) in Juvenile Spring- and Fall-Run Chinook Salmon of the Columbia River Basin

**DOI:** 10.3390/ani12151887

**Published:** 2022-07-24

**Authors:** Daniel G. Hernandez, Gael Kurath

**Affiliations:** 1 School of Aquatic and Fishery Sciences, University of Washington, Seattle, WA 98195, USA; dh38@humboldt.edu; 2 U.S. Geological Survey, Western Fisheries Research Center, 6505 Northeast 65th St., Seattle, WA 98115, USA

**Keywords:** infectious hematopoietic necrosis virus, Chinook salmon, virus shedding, shedding kinetics, Columbia River Basin, intraspecies variation

## Abstract

**Simple Summary:**

When a virus infects a host it reproduces in that host and then sheds from the host in order to find new hosts for more rounds of reproduction. Thus, virus shedding is a critical step in the host-to-host transmission cycles that allow a virus to spread across a landscape and persist over time. In Pacific salmon and trout the virus infectious hematopoietic necrosis virus (IHNV) causes significant disease, with up to 50% mortality in outbreaks in some conservation hatcheries. Chinook salmon have evolved as two distinct life-history types, referred to as spring- and fall Chinook salmon, and they are the most abundant host of IHNV in the Columbia River basin (CRB) of Washington, Oregon, and Idaho. Here we examined the timing and quantity of virus shedding from both spring-run and fall-run CRB Chinook salmon after controlled exposures to three IHNV strains representing different virus subgroups. We observed rapid shedding kinetics with similar timing for two virus strains in both host types. However, spring Chinook salmon shed much more virus from the UC subgroup than fall fish, suggesting that spring Chinook salmon may play a dominant role in the ecology and maintenance of IHNV in the CRB.

**Abstract:**

This investigation sought to characterize the shedding of infectious hematopoietic necrosis virus (IHNV) in two populations of Columbia River Basin (CRB) Chinook salmon (*Oncorhynchus tshawytscha*). Juvenile spring- and fall-run Chinook salmon were exposed by immersion to each of three IHN virus strains from the UC, MD, and L subgroups, and then monitored for viral shedding from individual fish for 30 days. Detectable quantities of UC, MD and L IHN virus were shed by a subset of fish from each host population (1–9 out of 10 fish total in each treatment group). Viral shedding kinetics were consistent, with a rapid onset of shedding, peak shedding by 2–3 days, and then a rapid decline to below detectable levels by 7 days’ post-exposure to IHNV. Intraspecies variation was observed as spring Chinook salmon shed more UC virus than fall fish: spring Chinook salmon shed UC virus in greater numbers of fish, with 22-fold higher mean peak shedding magnitude, 33-fold higher mean total virus shed per fish, and 900-fold higher total virus shed per treatment group. The L and MD viruses had comparable shedding at intermediate levels in each host population. All viral shedding occurred well before host mortality began, and shedding magnitude did not correlate with virulence differences. Overall, the greater shedding of UC virus from spring Chinook salmon, combined with low virulence, indicates a uniquely high transmission potential that may explain the predominance of UC viruses in CRB Chinook salmon. This also suggests that spring-run fish may contribute more to the ecology of IHNV in the CRB than fall-run Chinook salmon.

## 1. Introduction

Infectious hematopoietic necrosis virus (IHNV) is a rhabdoviral pathogen that causes significant disease impacts in several salmonid fish species [1,2]. Globally there are five major phylogenetic groups (genogroups) of IHNV that differ in host specificity and/or geographic range [3]. Three of these genogroups occur in North America, where L, M, and U viruses predominantly infect and cause disease in Chinook salmon (*Oncorhynchus tshawytscha*), rainbow trout and steelhead trout (freshwater and anadromous forms of *O. mykiss*), and sockeye salmon (*O. nerka*), respectively [4]. Geographically, the L genogroup occurs in California and the southern Oregon coast; the M genogroup is mostly restricted to the large Columbia River Basin (CRB) that drains much of Oregon, Washington, and Idaho; and the U genogroup occurs in the CRB and coastal watersheds of Washington, British Columbia, and Alaska. Phylogeographic studies of IHNV field isolates have defined several subgroups within these genogroups [5,6,7,8]. In the CRB nearly all M genogroup virus is in the MD subgroup [8,9]. Also in the CRB a subgroup of the U genogroup, UC, has evolved with a more generalist host pattern, infecting Chinook salmon, steelhead trout, and sockeye salmon, but typically not causing disease [7,9]. Thus, in the CRB watershed, IHNV subgroups UC and MD co-occur with distinct but overlapping host specificities and different disease impacts [9]. Chinook salmon in the CRB have a high prevalence of UC virus (>20%) without significant disease [10], and in California they have L viruses and experience severe disease outbreaks [6,11,12]. 

IHNV is predominantly transmitted fish-to-fish via waterborne horizontal transmission, especially among hatchery or farmed fish populations where disinfection of eggs is a common biosecurity practice that interrupts egg-associated transmission from parent to progeny [2]. The source of waterborne IHNV is virus shed from infected fish via urine, feces, or sloughing of mucus. Virus shedding has been used as a proxy for virus transmission potential, and an assay to quantify the shedding kinetics of IHNV from individual juvenile rainbow trout over the entire course of infection has been developed by Wargo and colleagues [13]. Shedding assays of several IHNV strains of varying virulence, in both single and mixed infections, have defined a remarkably consistent picture of IHNV shedding from infected rainbow trout as a process with a very rapid onset that peaks 2–3 days after exposure to virus and then wanes, often to levels that are undetectable by 5–7 days’ post-exposure (dpe) [13,14]. To date this assay has been applied to rainbow trout infected with M genogroup strains of IHNV. Here we have conducted the same shedding assay on individual juvenile Chinook salmon infected with IHNV strains from the UC and MD subgroups that co-occur in the CRB, and also an L genogroup strain known to cause disease in California Chinook salmon [11]. 

In previous studies we investigated susceptibility of Chinook salmon to infection and disease after exposure to L, UC, and MD strains of IHNV [15,16]. Pacific Northwest Chinook salmon occur as numerous sub-populations that vary in both genetics and life-history phenotypes [17,18,19,20]. The dominant life-history phenotypes are referred to as fall-run and spring-run Chinook salmon, which vary in many traits including spatial ranges and seasonal timing of both juvenile out-migration and adult return migration for spawning. Experimental infections of juvenile Chinook salmon from four CRB populations, including fall- and spring-run fish from upper and lower basin sources, revealed little intra-species variation in host susceptibility to mortality, with the UC and MD viruses having very low virulence and only the L virus causing significant disease [16]. Similarly, there was little intra-species variation in several measures of infection, with the UC and MD viruses being very similar in infectivity [16]. This is in contrast to the notable asymmetry of IHNV prevalence in CRB Chinook salmon, which is 82–88% UC and 12–18% MD for both spring-run and fall-run fish [16]. 

The objectives of the current study are (1) to define the IHNV shedding kinetics in Chinook salmon, for comparison with the rapid shedding kinetics reported for M genogroup IHNV in rainbow trout, (2) determine if shedding kinetics or magnitude differ for L, UC, or MD virus infections of Chinook salmon, and (3) determine if there is intraspecies variation in IHNV shedding from fall-run and spring-run CRB Chinook salmon. Our goals are to expand the current fundamental knowledge of IHNV shedding, and to provide novel insights into possible drivers of UC and MD IHNV ecology in the CRB.

## 2. Materials and Methods

### 2.1. Experimental Fish Populations

The spring-run and fall-run Chinook salmon populations selected for this study were tested previously for susceptibility to infection and mortality after exposure to UC, MD, and L strains of IHNV and were described in [16]. The spring-run Chinook salmon were from the Methow River population (No. 28 in Narum et al. [18]) reared at the Winthrop National Fish Hatchery (U.S. Fish and Wildlife Service). The fall-run fish were from the Cowlitz River population (No. 1 in Narum et al. [18]), reared at the Cowlitz Hatchery (Washington Department of Fish and Wildlife). For each population a total of 2000 eyed eggs (developing embryo), sourced from a minimum of 12 parental spawning pairs, were incubated and hatched at the USGS Western Fisheries Research Center (WFRC) wet laboratory in Seattle, WA where they were reared to approximately 1 g at a constant temperature of 10 °C. Juvenile Chinook salmon were fed a semi-moist pellet diet (BioOregon) at a rate of 1.0–2.0% body weight per day. All fish rearing and experimental exposures were conducted using single-pass, flow-through, sand-filtered and UV-treated freshwater from Lake Washington.

### 2.2. Experimental Virus Strains

IHN viruses are members of the species Novirhabdovirus salmonid, genus Novirhabdovirus, Family Rhabdoviridae [21]. Three IHNV strains used here, FR0031, RB1, and QTS07, represented the LII, UC, and MD subgroups of IHNV, respectively, as described previously [16]. They are known to have high virulence in Chinook salmon, sockeye salmon, and steelhead trout, respectively [16,22,23], and each has a specific midG genotype that is commonly detected among field isolates within its subgroup [8,11,16]. Virus stocks were amplified in the epithelioma papulosum cyprinid (EPC) cell line [24,25] using minimal essential media with 10% fetal calf serum, and quantified by plaque assay in EPC cells as previously described [26].

### 2.3. Virus Exposure and Monitoring

Viral challenges and subsequent monitoring were conducted at a constant water temperature of 10 °C to mimic average conditions observed throughout the CRB [20]. To assess the shedding kinetics of IHNV in spring- and fall-run Chinook salmon, juvenile fish (1 g) from each host population were exposed to L, UC and MD strains of IHNV and held in individual tanks for collection of shed virus samples over time as previously described [13,14]. For each host type 30 fish were exposed to each virus strain by static immersion for 1 h in 1 L of water containing virus at a dose of 2 × 10^5^ plaque forming units (PFU) mL^−1^. A group of 30 fish was also exposed to virus-free media as a negative control. Water flow was then resumed and fish were rinsed in flowing water at a total volume of 5 L per tank for 1 h. Following the rinse, 10 fish from each viral treatment (6 from the virus-free negative control) were placed into individual 1.5 L tanks in a tower rack system (Aquatic Ecosystems) with water flowing to each tank at approximately 200 mL min^−1^. A 1.4 mL sample of water was collected from each tank immediately after fish distribution. Water flow was then turned off for 23 h to allow for accumulation of shed virus. A constant temperature of 10 °C was maintained by circulating temperature-controlled water around the tanks. Following the 23 h static period, water was sampled from each tank and flow was then turned on for 1 h to flush out any shed virus in the water [13,14]. Cycles of 23 h of static hold, followed by water sampling, and then a 1 h flush in flowing water were repeated daily, and samples were collected 0, 1, 2, 3, 4, 5, 7, 8, 10, 14, 22, and 30 days’ post exposure (dpe). Water samples were stored at −80 °C for later RNA extraction.

After 10 fish were removed from each immersion challenge treatment group and placed into individual tanks, the remaining 20 fish in each group were held together in a single tank of flowing water and monitored daily for mortality over the course of the 30-day experiment. These conditions provide benchmark data to facilitate comparison of mortality with that reported previously for the same treatment groups using the same conditions in triplicate tanks [16]. At the end of the 30-day observation period, all surviving fish from both batch and individual tanks were euthanized using buffered Tricaine Methanesulfonate (Western Chemical, Inc., Ferndale, Washington, DC, USA) at a concentration of 240 mg L^−1^.

### 2.4. RNA Extraction and Quantification of Shed Viral RNA

Viral RNA was extracted from 200 ul of each water sample using the QIAamp cador Pathogen Mini Kit (QIAGEN), following manufacturer’s recommendations. Viral RNA in 100 uL of AVE buffer (QIAGEN) was assessed for quality and concentration by spectrophotometry before complementary DNA (cDNA) was synthesized using M-MLV reverse transcriptase with random hexamer primers [27]. A standard volume of 5 µL of RNA was used in each cDNA reaction, and the final 20 µL of cDNA was diluted 1:2 by adding 20 µL of RNase-free water before storage at −80 °C.

Viral RNA was quantified using the universal IHNV N gene reverse transcriptase real-time PCR (RT-rPCR) assay as previously described [28]. Briefly, 5 µL of each diluted cDNA sample was combined with forward and reverse primers, TaqMan FAM-labeled probe for the IHNV N gene, VIC -labeled probe for the artificial positive control (APC) and amplified on an Applied Biosystems ViiA7 real-time PCR machine. APC plasmid DNA was linearized and used to construct a standard curve (5 × 10^7^ to 5 DNA copies) with which to quantify the absolute copy number of viral RNA (Purcell et al., 2013). Each sample was run in duplicate wells and interpreted as positive only when amplification was detected in both replicates within 40 cycles. Samples whose duplicate well results were not in consensus were considered suspect, requiring further analysis. For suspect samples, new cDNA was synthesized from RNA and assayed again with the universal IHNV N gene RT-rPCR assay in 4 independent qPCR reactions, each in one well. Suspect samples whose secondary RT-rPCR assay resulted in two or more positive replicate wells were confirmed positive and included in data analysis as positive samples. Suspect samples whose secondary RT-rPCR assay resulted in one or less positive replicate wells were considered negative.

The analytical sensitivity of the IHNV RT-rPCR assay was determined based on the PCR efficiencies observed for the APC plasmid DNA standard curves for all assays included in this analysis [28]. The reaction efficiencies varied from −3.46 to −3.64 and the y-intercept values varied from 40.0 to 41.5, indicating similar limits of detection across the IHNV RT-rPCR assays. The theoretical detection limit of the IHNV N gene RT-rPCR assay based on the standard curves was 566.9 viral RNA copies per ml of H_2_O (2.75 log_10_ RNA copies mL^−1^), referred to hereafter as shed viral load. Note that calculated values for total shed viral load per fish are also reported here as log_10_ RNA copies mL^−1^, but the tanks that held individual fish had volumes of 1500 mL of water. Therefore, assuming a uniform concentration in the tanks, the actual totals shed per fish would be 1500× the amount shed per fish reported here in units of log_10_ RNA copies mL^−1^.

### 2.5. Statistical Analyses

#### 2.5.1. Number of Fish Shedding over Days 2–5 (Fish Shedding-Days)

The sum of the numbers of fish shedding daily over the shedding period between days 2–5 in each viral treatment group (L, UC and MD IHNV), referred to as the number of fish shedding-days, was analyzed using Fisher exact tests on a contingency table of the sum of the numbers of fish shedding detectable virus on each day versus the total number of fish tested for the four days (*N* = 40). This test was performed separately for each host population between viral treatments, constituting six tests. This was also performed separately for each viral treatment between host populations, constituting three additional tests. To account for multiple pairwise comparisons, a Bonferroni correction of the 0.05 significance level was performed by dividing it by the total number of comparisons (0.05/9) resulting in α = 0.005.

#### 2.5.2. Viral Shedding Kinetics

Data generated from the virus-shedding assays were used to test hypotheses relevant to IHNV shedding kinetics in CRB Chinook salmon. Measures of viral shedding including mean day of peak shedding, mean peak quantity, and mean total quantity of virus shed per fish were compared between experimental groups under the null hypothesis of equality. One-way Analysis of Variance (ANOVA) was used to test for the equality of means, where rejection of the null hypothesis was based on the calculated F statistic being equal to or greater than the critical value [29]. Tukey’s Honestly Significant Difference (HSD) a posteriori test for multiple comparisons was used to identify which treatment groups differed. All statistical analyses were conducted in GraphPad Prism software [30]. From the host perspective, one-way ANOVA was robust in concluding whether host population had an effect on the shedding kinetics of IHNV. From the virus perspective, hypothesis testing was robust in determining whether virus strains had the same effect on viral shedding.

#### 2.5.3. Survival of Experimental Fish Populations

To assess the influence of viral treatment on host survival, survival analysis was conducted using the Kaplan-Meyer method. Survival curves were constructed for each group of 20 Chinook salmon held together in batch and also for treatment groups of 10 fish held in isolation and monitored for viral shedding. Survival curves were compared between viral treatment groups, within and across host types, using a log-rank (Mantel-Cox) test in GraphPad Prism [30].

## 3. Results

### 3.1. Number of Fish Shedding Virus

**Figure 1:**
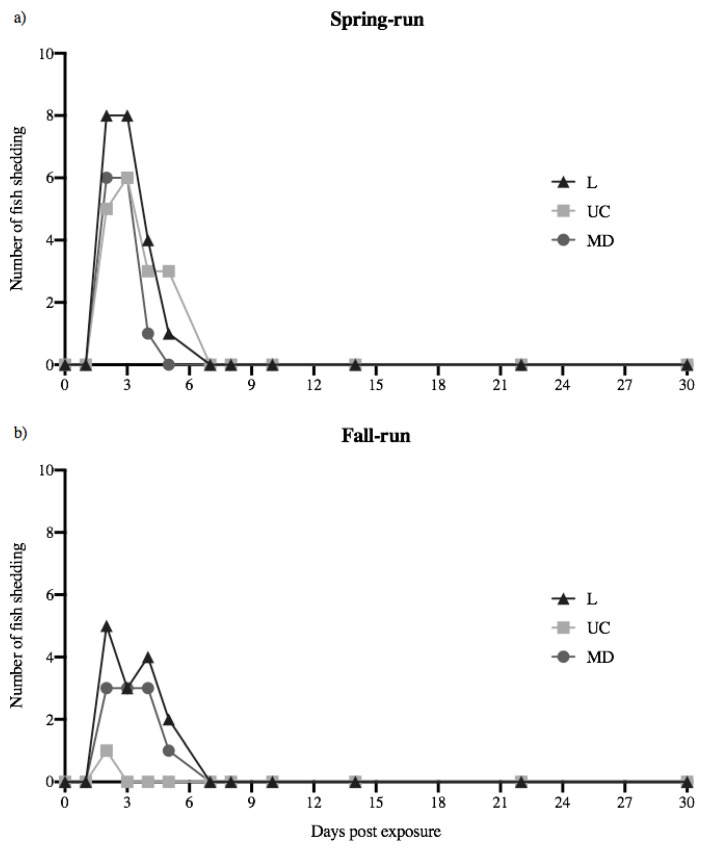
Daily number of fish shedding detectable virus over the 30-day course of infection. Spring- (**a**) and fall-run (**b**) Chinook salmon shedding L, UC and MD IHN virus per day, out of 10 fish in each treatment group. No fish in the virus-free treatment (negative control) shed detectable virus over the course of the 30-day experiment.

Juvenile spring- and fall-run Chinook salmon each shed detectable quantities of L, UC and MD IHNV, with numbers of fish shedding in each treatment group over time illustrated in Figure 1. Across all 60 virus-exposed fish, 34 shed detectable virus over the 30-day course of infection. The number of fish shedding ranged from 1–9 out of 10 fish in each treatment group. No detectable virus was shed at time zero or 24 h following exposure. Viral shedding was first detected at 48 h (2 days), and last detected 5 days’ post-exposure (dpe). No viral shedding was detected in any treatment group at time points sampled on days 7, 8, 10, 14, 22, and 30. Over the course of the 30-day observation period, no detectable virus was shed in mock-exposed fish (negative control treatment) at any time. For each host population the L treatment group had the highest number of fish shedding virus. The total numbers of fish shedding L, UC and MD virus were higher in spring-run Chinook salmon (6–9 fish out of 10) relative to fall-run fish (1–7 out of 10). Most notably, far more spring-run fish shed UC virus relative to fall-run fish. Over the 30-day course of infection, UC virus was shed by 6 of 10 spring-run fish, but only by one fall-run fish, for only one day.

The numbers of fish shedding daily between days 2–5 in each viral treatment group (L, UC and MD IHNV for each fish host population) were analyzed as “fish shedding-days”, calculated as the sum of the total numbers of fish shedding detectable virus on each of the four days versus the total number of fish tested for the four days (*N* = 40), with a corrected α = 0.005 (see methods). For spring-run Chinook salmon there were no significant differences between the numbers of fish shedding-days for L, UC and MD virus treatments (*p* > 0.005). Within fall-run Chinook salmon, the numbers of fish shedding-days for L and MD virus, or MD and UC virus, were not significantly different (*p* > 0.005). However, for fall-run Chinook salmon the number of fish shedding-days for UC virus (1 out of 40) was significantly different than for L virus (14 out of 40) (*p* = 0.0003). In comparisons across fish populations the numbers of spring- and fall-run fish shedding-days for L virus, or for MD virus, showed no significant differences (*p* > 0.005). However, for UC virus the numbers of fish shedding-days for spring-run fish (17/40) and fall-run fish (1/40) were significantly different (*p* = 0.0001).

### 3.2. Kinetics and Magnitude of Viral Shedding

**Figure 2:**
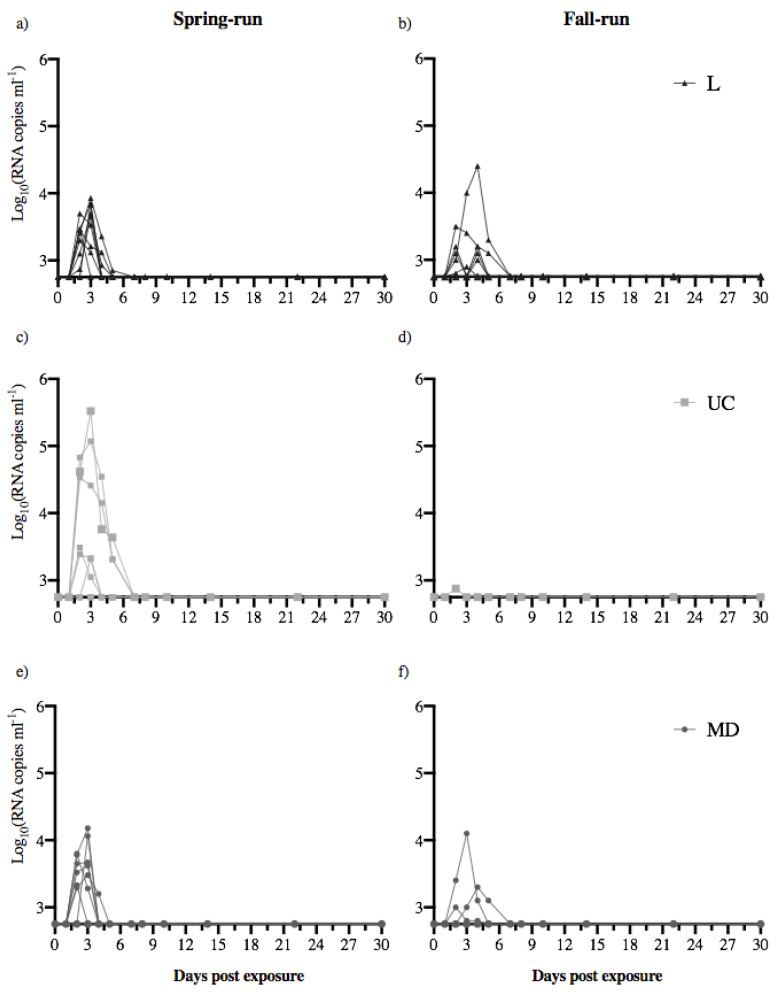
Daily quantity of virus shed by individual fish. Each panel shows the shedding kinetics of individual fish over the 30-day course of infection. Daily quantities of L (**a**,**b**), UC (**c**,**d**) and MD virus (**e**,**f**) shed from spring- (**left**) and fall-run Chinook salmon (**right**) are reported as log_10_ virus RNA copies mL^−1^. The detection limit of the universal N gene IHNV reverse transcriptase real-time PCR (RT-rPCR) assay (2.75 log_10_ RNA copies mL^−1^) is reflected as the base of the *y*-axes. No fish in the virus-free treatment (negative control) shed detectable virus.

The kinetics of virus shedding from individual fish is illustrated in Figure 2. Across all virus-exposed treatment groups, shedding from a subset of fish increased rapidly, with the peak amount of shed virus occurring between days 2–3 in 88% of the fish (44% day 2, 44% day 3). Viral shedding began to decrease from day 3 forward, such that by day 7, no fish shed detectable virus. The total number of days an individual fish shed detectable virus ranged between 1 and 5 days (Figure 2), with an overall mean of 2.24 days shedding.

**Figure 3:**
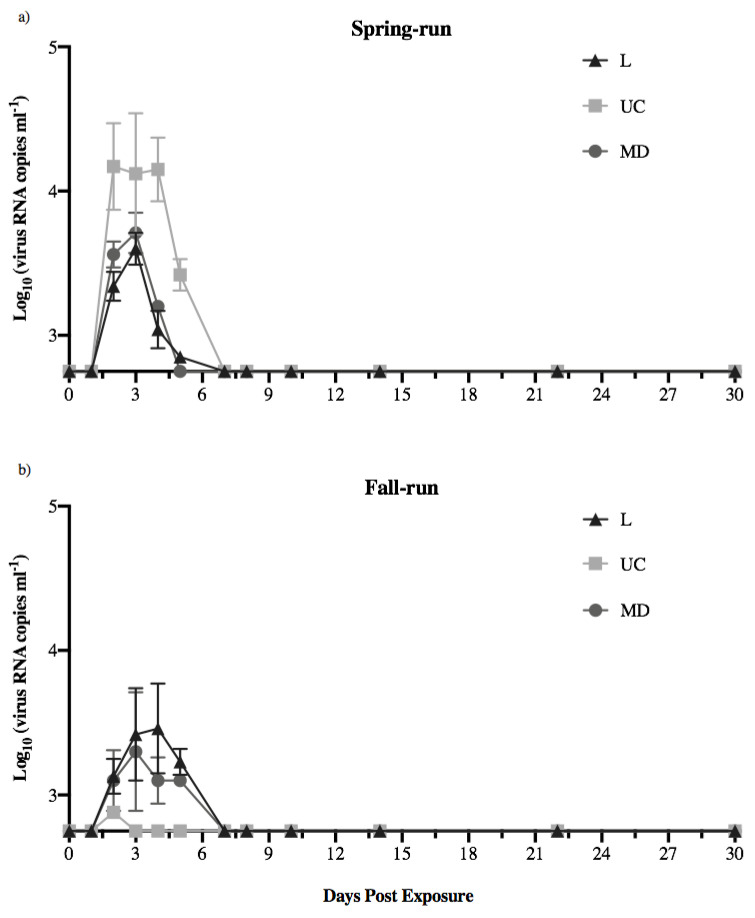
Mean shedding kinetics of L, UC and MD IHNV in (**a**) spring- and (**b**) fall-run Chinook salmon of the CRB. Data are mean (±1 standard error) virus quantities shed daily for all fish that shed virus on each day in each treatment group. The detection limit of the universal N-gene IHNV rPCR assay (2.75 log_10_ RNA copies mL^−1^) is reflected as the base of the *y*-axes.

Mean shedding kinetics for fish that shed detectable virus within each treatment group are shown in Figure 3. As noted above, shedding of virus increased rapidly across all viral treatments, reaching the highest observed quantities between days 2–3, and decreasing thereafter. Within and across host populations, the shedding kinetics of the L and MD virus were comparable over time. Within spring-run Chinook salmon, shedding of the UC virus was approximately 1 log higher over time than observed for the L and MD viruses (Figure 3a). In contrast, within fall-run fish the single fish that shed UC virus had a much lower peak magnitude than the L or MD shedding peaks (Figure 3b). Among all treatment groups the UC virus was shed by spring-run fish in the highest mean quantities over time.

**Figure 4:**
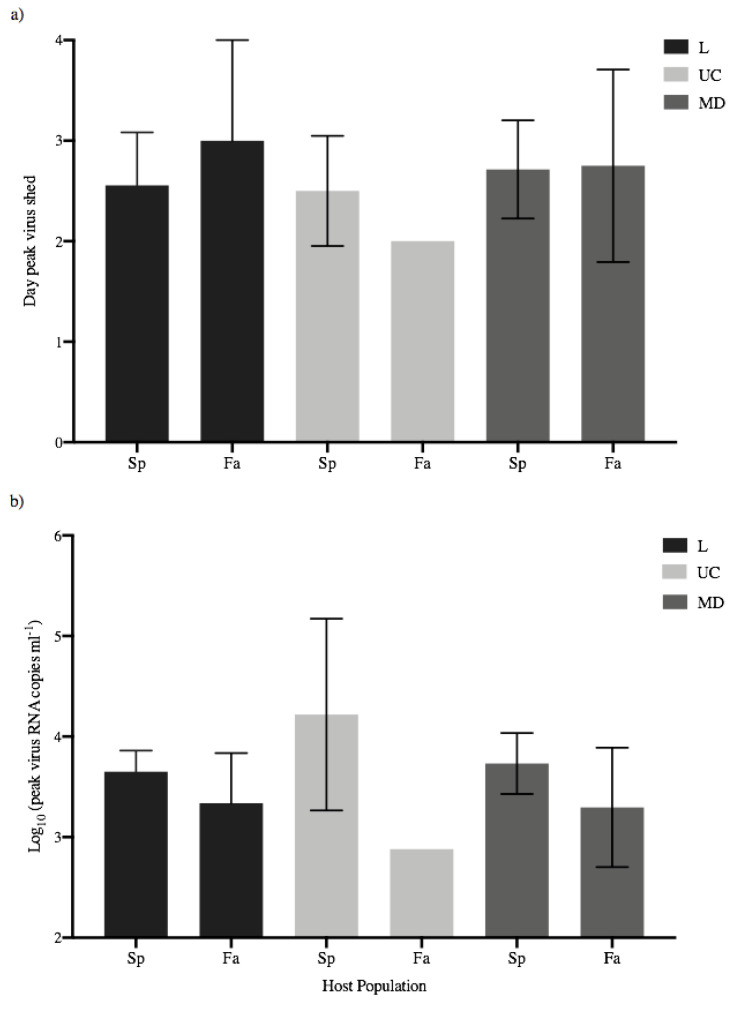
Peak shedding patterns of individual spring- (Sp) and fall-run (Fa) Chinook salmon. (**a**) Mean day (±1 standard error) of peak shedding of L, UC and MD viruses in individual spring- and fall-run fish. (**b**) Mean quantity (±1 standard error) of peak virus shed in fish within each treatment group, irrespective of day.

Peak viral shedding for each of the 34 fish that shed detectable virus was determined as the data point showing the highest shed viral load for each fish. The mean day of peak shedding for each treatment group varied between day 2 and 3, and there were no significant differences between IHNV treatments or across host populations (ANOVA, *p* > 0.05) (Figure 4a). The mean quantity of peak virus shed in each treatment group ranged from 2.88–4.22 log_10_ viral RNA copies mL^−1^ (Figure 4b, Table S1). The highest and lowest peak-shedding values were both for UC virus, with a 22-fold difference between the highest mean peak, for UC in spring-run fish, and the lowest mean peak, for UC in fall-run fish. Mean peak-shedding values for L and MD virus in both fish populations were at intermediate levels that were much more consistent, ranging within a 2.7-fold difference between 3.30–3.73 log_10_ viral RNA copies mL^−1^. In comparisons of different viruses within spring fish the mean peak for UC was 3–4 fold higher than for MD and L viruses. In contrast, fall fish had lower peak shedding values overall, and mean peak shedding quantities for L and MD viruses were 2–3 fold higher than for UC (again note UC shed from only one fall-run fish) (Figure 4b, Table S1). Despite the observed variation there were no significant differences in mean peak quantities of shed virus within or across host populations (ANOVA, *p* > 0.05). The lowest peak magnitude in the single fall-run Chinook salmon that shed UC virus did not overlap the error bars for the mean peak values in several other treatment groups (Figure 4b), and it was 22-fold lower than UC in spring-run fish, but the difference was not significant, likely due to inherent limitations in statistical analysis of a single data point.

### 3.3. Total Virus Shed by Individual Fish

**Figure 5:**
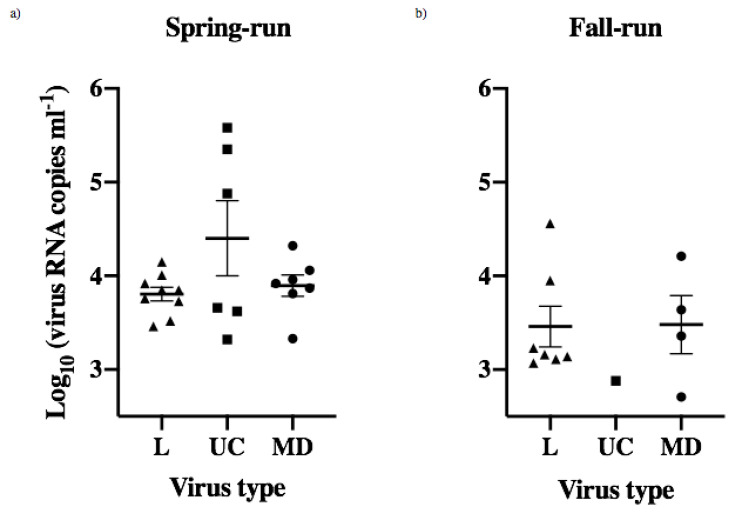
Total quantities of L (triangles), UC (squares) and MD (circles) virus shed by individual juvenile spring- (**a**) and fall-run (**b**) Chinook salmon over the 30-day course of infection. Means (±1 standard error) of total quantities of virus shed by individual fish in each treatment group were calculated for only those fish that shed virus over the 30-day course of infection.

Beyond peak shedding values, the broader IHNV transmission potential in individual Chinook salmon can be determined by the total quantities of L, UC or MD virus shed by each juvenile fish over the 30-day course of infection, which in this data set is the total shed on days 2–5. The mean total quantities for fish that shed virus in each treatment group (Figure 5) ranged from 2.88–4.40 log_10_ viral RNA copies mL^−1^ (Figure 4b, Table S1), with the highest and lowest values representing a 33-fold higher mean total shed per fish in the spring fish UC group compared with the fall fish UC group. The mean total shed for MD and L viruses was nearly equal in each host (within 1.2-fold), and spring fish shed 2.2–2.6 fold more total MD or L virus per fish than fall fish. There were no significant differences for the mean total virus shed per fish within or across host populations (ANOVA, *p* > 0.05). Again, although not significant, the single fall-run fish that shed UC virus had a total well below the standard error range for all five other treatment groups (Figure 5, Table S1).

### 3.4. Total Virus Shed by Each Treatment Group

**Figure 6:**
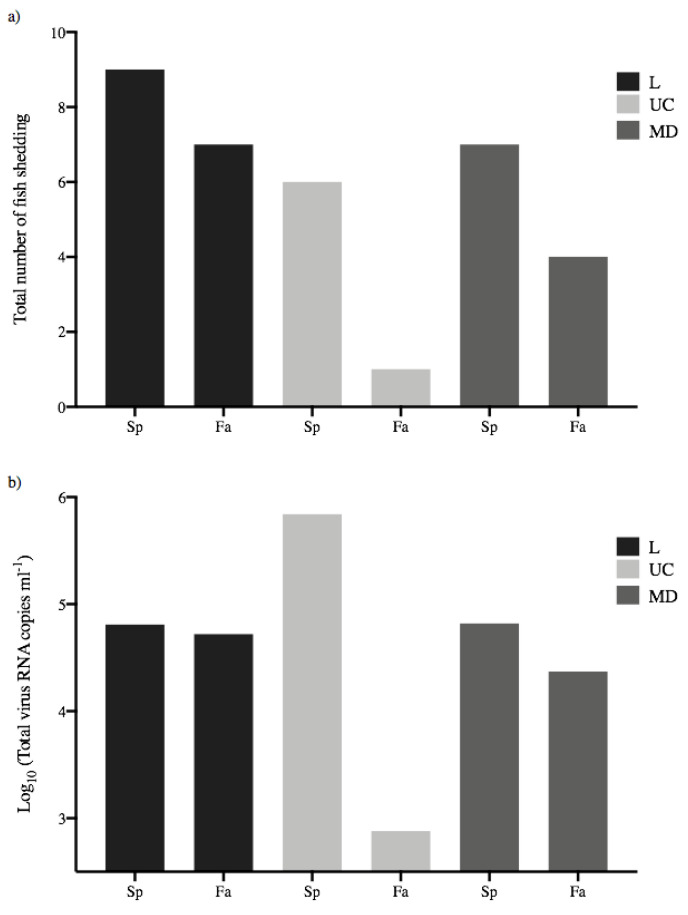
Population-based transmission potential of each treatment group. (**a**) Total number of spring- (Sp) and fall-run (Fa) Chinook salmon shedding L, UC and MD IHN virus over the 30-day course of infection. (**b**) Total virus quantity shed by all fish per treatment group.

The population level transmission potential of each treatment group can be determined by considering the total amount of virus shed by all virus-exposed fish within each group [13]. Figure 6a shows the total number of fish shedding virus within each treatment group, and Figure 6b shows the total amount of virus shed by all fish in each group. Values for total virus shed by each group ranged from 2.88–5.84 log_10_ viral RNA copies mL^−1^, with the lowest and highest values again representing UC virus shed by fall- or spring-run fish, respectively. This indicated that, on a population basis, the spring-run UC treatment group shed more than 900-fold more virus than the fall-run UC group, due to the combined effects of higher numbers of fish shedding (Figure 6a) and higher mean total shedding quantities (Figure 5) for UC in spring-run fish. The total quantities of L or MD virus shed by either spring- or fall-run treatment groups were again intermediate and more consistent, within a 3-fold range between 4.37–4.82 log_10_ viral RNA copies mL^−1^. In comparisons of the total group shedding for viruses within each host, spring-run fish shed approximately 10–fold more UC than MD or L virus. Fall-run fish shed 70-fold more L than UC, and 31-fold more MD than UC virus (but again note UC shed from 1 fall-run fish). It is interesting to observe that for spring-run Chinook salmon, although more fish shed L virus (9/10 fish) compared to UC virus (6/10 fish), there was 10-fold more UC than L virus shed on a population level, due to the sustained higher mean viral loads of UC shedding. Because the total amount of virus shed for each treatment group is a single number there was no statistical analysis.

### 3.5. Correlation of Duration and Total Magnitude of Shedding

**Figure 7:**
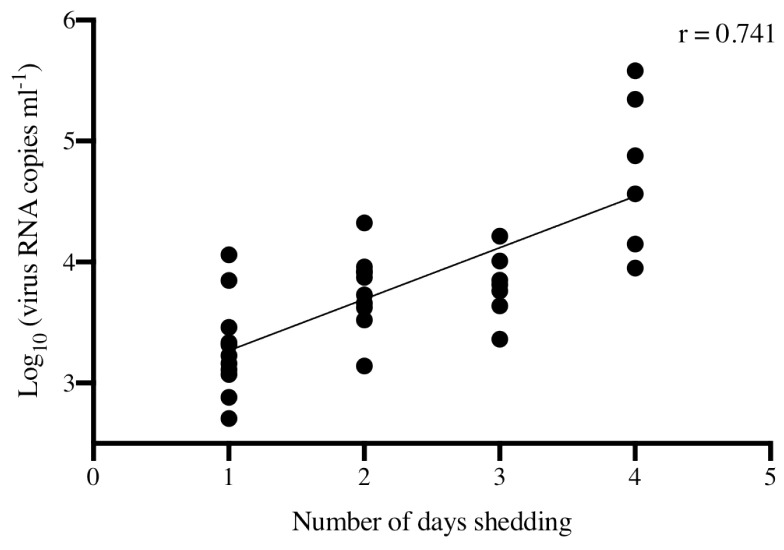
Correlation between the total quantity of virus shed by individual fish and the total number of days shedding detectable virus, irrespective of virus strain or fish population. Pearson r correlation coefficient is reported (*p* < 0.0001).

Sum total quantities of virus shed by individual fish and the total number of days each fish shed virus were assessed for correlation, irrespective of virus strain and host population (Figure 7). The Pearson r coefficient reported (r = 0.741) indicated that a statistically significant correlation (*p* < 0.0001) existed between the total quantity of virus shed by individual Chinook salmon and the total number of days a fish sheds.

### 3.6. Survival of Experimental Fish Populations

**Figure 8:**
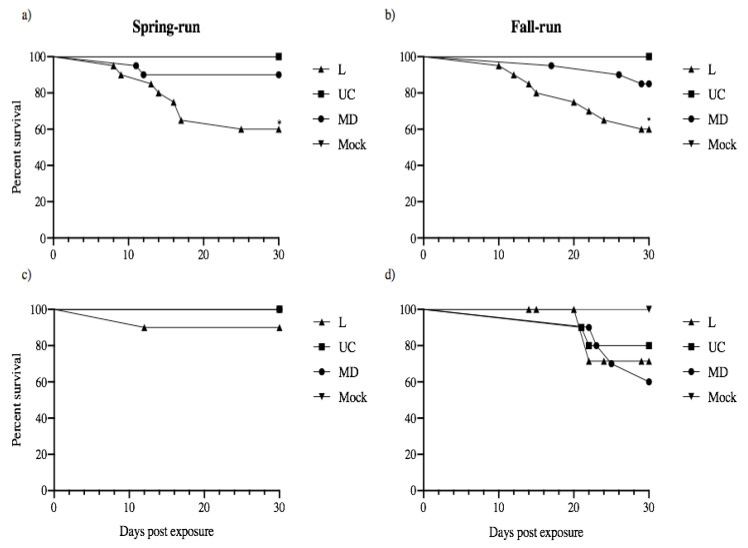
Daily cumulative percent survival of juvenile spring- (**a**,**c**) and fall-type (**b**–**d**) Chinook salmon exposed to L, UC and MD strains of IHNV or virus-free media (Mock). Experimental host populations were monitored for mortality over the course of 30 days. 20 fish from each treatment group remained together in batch following the immersion challenge (**a**,**b**), whereas 10 of the initial thirty fish exposed were isolated to quantify the viral shedding of each individual (**c**,**d**). Survival curves were constructed for each group of 20 fish per batch treatment and those fish individually monitored for viral shedding. Asterisks indicate that fish in the positive control L genogroup IHNV treatment had significantly lower survival than batch-held fish in the other viral strain treatments (*p* < 0.001). No significant reductions in survival were observed in batch-held or isolated fish exposed to the UC and MD virus treatments.

Following exposure to L, UC and MD strains of IHNV by immersion challenge, 20 fish from each original challenge treatment remained together post viral exposure to provide an indication of batch mortality that will facilitate comparison with previously published data on infections and virulence [16]. In addition, juvenile spring- and fall-run Chinook salmon that were held in isolation and sampled for shed virus were also monitored for mortality over the course of 30 days. Thus, for each treatment group survival curves were constructed for the group of 20 fish held in batch and for those fish individually monitored for viral shedding (Figure 8). No mortality was observed in any mock-exposed fish (virus-free treatment). For fish held in batch the positive control L IHNV treatments in both fish populations had significantly lower survival than fish in the other viral treatments (*p* < 0.001). Mortality began on days 8–10 and occurred more rapidly in spring-run than fall-run fish, reaching approximately 40% mortality in each host type by 30 dpe. No significant reductions in survival were observed in batch-held fish exposed to the UC and MD virus treatments. Across fish held in isolation, mortality first occurred at 12 dpe in spring-run fish, and 21 days for fall-run fish. Final mortality in groups of 10 fish held in isolation was 10% for spring-run fish exposed to L virus, and 22–40% for fall-run fish exposed to each of the three viruses. Although fall-run fish in isolation had more mortality events than spring-run fish in isolation, no statistically significant reductions in survival were observed within or across host populations held in isolation following exposure to L, UC and MD virus treatments. All viral shedding occurred well before the onset of host mortality in any group.

## 4. Discussion

In 2017 Wargo and colleagues first described a novel experimental assay that quantified IHNV shedding kinetics based on shedding profiles from multiple individual juvenile rainbow trout exposed to each of two M group strains of virus [13]. Daily shedding of virus was determined over a twelve day course of infection by quantifying the amount of viral RNA shed during a 23 h static period each day. They observed a peak of IHNV shedding by day 2 post-exposure, followed by a rapid decline by day 5 to a plateau approximately 2-log lower than peak shedding values. Their summary was that “viral shedding for IHNV is a very acute process that begins with a rapid peak after exposure to virus and tapers off quickly to a post-peak period of lower shedding magnitude that can extend for at least 12 days”. In a subsequent study they found very similar IHNV shedding kinetics for additional M genogroup strains in single and mixed infections of juvenile rainbow trout and reported a positive correlation between virulence and total shedding over a longer infection course of 30 days [14]. These first two studies defined consistent shedding kinetics for several M IHNV strains in rainbow trout, and the authors noted that “expanded studies with different IHNV genotypes and different hosts will be essential to assess if shedding kinetics is a variable viral phenotype” [13].

Here we expand the investigation of IHNV shedding kinetics to a different host, Chinook salmon, and use the same experimental assay to test IHNV genotypes from all three of the major IHNV genogroups in North America, U, M, and L. From an ecological perspective, we specifically tested IHNV genotypes from the UC and MD subgroups that co-occur and predominate in the CRB, and we included an L genotype as a positive control known to be highly adapted to Chinook salmon in California. We also investigated possible intraspecies variation by comparing shedding kinetics between two genetically diverse populations of CRB Chinook salmon that represent the fall-run and spring-run life history phenotypes that predominate in the CRB. We found similar kinetics of shedding for all three IHNV strains in both Chinook salmon populations, with a rapid peak by 2–3 dpe, followed by a decline to levels below detection by 7 dpe. These results are consistent with the rapid kinetics reported for M viruses in rainbow trout [13,14]. This suggests that IHNV shedding kinetics may be a consistent phenotype for viruses from different genogroups and in different host species that co-evolved in the field under different conditions. The shedding assays here were done at 10 °C to mimic general conditions in the CRB, whereas the previous studies were done at the 15 °C temperature typical for rainbow trout farms of southern Idaho [13,14]. Also the Chinook salmon populations tested here were from conservation hatcheries, where the annual spawning cycles are substantially slower than temporally altered spawning cycles used in commercial trout farms to provide a year-round supply of juvenile fish [5,14,31].

Beyond the consistent general kinetics profiles we found several interesting differences among treatment groups in comparisons of the numbers of fish shedding, the magnitude of peak shedding, and the total amount of virus shed in each treatment group. Looking first at intra-species variation, the biggest difference was in the shedding of UC virus by the two Chinook salmon populations. Compared with fall-run fish, spring-run fish infected with UC virus shed in higher numbers (6/10 fish versus 1/10 fall-run fish), had higher mean peak shedding (22-fold), higher mean total shed per fish (33-fold), and higher total virus shed per treatment group (900-fold). This indicates a dramatic difference in the ability of spring- and fall-run fish to shed UC virus, with spring Chinook shedding much more virus both individually and on a population basis, despite no significant difference in UC related mortality between run-types. This is the first observation of a strong intraspecies difference in an IHNV fitness trait [16]. This is particularly interesting in contrast to the similarity in susceptibility to infection observed in our previous studies, where infectivity (quantified by determining the infectious dose needed to infect 50% of the fish, ID50s) and viral loads of UC virus at 3 or 7 days’ post exposure did not differ for four populations of spring and fall Chinook salmon, including the two populations tested here [15,16]. This indicates that susceptibility to infection and within-host infection levels are not always strongly correlated with the ability to shed virus, as often assumed.

In comparing the three virus strains, among spring Chinook salmon treatment groups the shedding of UC virus was 3–4 fold higher than MD or L virus by mean peak quantities or mean total quantities per fish, and it was 10–11 fold higher by total amount shed per group. This suggests an overall higher capacity of spring-run fish to shed UC virus, despite controlled exposure to the same virus dose, and previous data indicating similar UC infection levels in spring- and fall-run fish [16]. It is especially interesting that there is more UC virus shed than L in spring-run fish, despite the fact that a higher number of the fish shed L virus. Although the L virus was included as a positive control that is highly adapted to Chinook salmon in California, the majority of California Chinook salmon are fall-run populations, which did not show the high level of UC virus shedding here. In our study fall Chinook salmon shed 3–4 fold more L or MD virus than UC virus by peak or total quantities per fish, and 31–70 fold more UC virus than L or MD by total virus shed per group.

All detectable virus shedding in the current study occurred well before mortality began, as previously reported for M virus shedding in rainbow trout [14], and for the related rhabdovirus, viral hemorrhagic septicemia virus (VHSV), shed from Pacific herring (*Clupea pallasii*) [32]. Mortality levels observed here for Chinook salmon held in batch were nearly identical to results from our recent studies [15,16], with approximately 40% mortality for groups exposed to L virus, and little or no mortality (no significant difference from the mock controls) for UC and MD groups. This indicates that challenge severity was similar between our previous studies and the work presented here, allowing us to integrate the current virus-shedding results with previous results of virulence and infectivity assays with the same viruses in the same CRB Chinook salmon populations [16]. A conclusion from that work was that the low virulence of UC and MD IHNV in CRB Chinook salmon is not driven by the inability of these viruses to enter juvenile fish, but rather by the ability of the juvenile fish to control UC and MD viral infections. Here we find that although UC virus has low virulence, it has the highest shedding potential in spring Chinook salmon. Also L and MD virus shedding is comparable in both spring and fall Chinook salmon, despite their high and low virulence phenotypes, respectively. These observations suggest that although a correlation between virulence and shedding was found for M strains of variable virulence in rainbow trout [14], this correlation does not extend uniformly to interactions of L, UC, and MD virus strains in Chinook salmon. The low virulence of UC virus combined with the high shedding phenotype in spring Chinook salmon would be highly advantageous for overall viral fitness.

The fact that only one fall Chinook salmon shed UC virus is a fascinating observation, but also a caveat to the current study, because all data for that treatment group are based on a single fish. As noted in the results section, this was a limitation for statistical analyses of this data set, where the most obvious difference observed, between spring and fall fish infected with UC, was not significant by most measures. It is therefore important to consider our level of confidence in the one positive data point for UC virus in spring Chinook salmon. That data point was from a fish that was originally positive in only one out of two duplicate wells of the RT-rPCR plate. By our standard procedures (see methods) this is considered suspect, which led to repeated testing of the RNA by synthesis of new cDNA used as template for four replicate rPCR reactions [28]. Two of the four repeated reactions were positive, which is defined as a positive, but this clearly indicated that the level of viral RNA in this sample was near the borderline of detection for the assay. A second fish in the same treatment group was also initially positive in only 1 of 2 wells, but in repeat testing only one of the four wells was positive, which was defined as a negative test. The finding of two suspect samples within the treatment group allows us to rule out the possibility that somehow this group of fish was not actually exposed to virus, and based on the rigorous testing we are confident that the one sample was truly virus positive. As a further observation the high level of UC shedding in the spring Chinook salmon was based on six positive fish, but their total shedding quantities were not uniform, with three fish exceptionally high and three fish lower, in the range of spring-run fish exposed to other viruses (Figure 5). These caveats, and the variable numbers of fish shedding in treatment groups of 10 fish, suggest that future confirmation of these results with larger numbers of fish would be valuable.

The acute timing of IHNV shedding observed here for Chinook salmon, and previously for rainbow trout [13,14], is interesting in comparison with other reports for shedding kinetics of aquatic pathogens. Garver et al. [33] found slower shedding kinetics for UP genogroup IHNV from individual or batch-held Atlantic salmon, with onset of shedding on day 8–9 and peak shedding at 16–26 days after immersion challenges. This demonstrates that IHNV shedding kinetics are not always as rapid as observed here, but there were many variables between the two studies including host species, host size (>300 g for the Atlantic salmon), virus genogroup and lower challenge dose. More protracted shedding kinetics were also reported for the fish rhabdovirus VHSV in juvenile muskellunge (*Esox masquinongy*), with high shedding through 5 weeks after the immersion challenge [34]. A bacterial pathogen in Chinook salmon, *Renibacterium salmoninarum* (the causative agent of bacterial kidney disease) also had much slower shedding kinetics than IHNV, with peak shedding at 22–35 days’ post-exposure [35,36]. The shedding kinetics most similar to IHNV in rainbow trout and Chinook salmon are described by Hershberger et al. for VHSV genotype IVa shed from Pacific [32,37]. They report slightly slower kinetics with the onset of VHSV shedding on day 3–5, peak shedding on days 6–10, and a decline to undetectable levels by day 16 post-exposure. However, they also describe a correlation of more rapid kinetics with higher challenge doses, and they used lower immersion challenge doses of virus than we used here (generally 10^3^ pfu/mL or lower, whereas we used 2 × 10^5^ pfu/mL). Therefore, it is uncertain whether IHNV shedding kinetics in salmonids are actually faster than those of VHSV in Pacific herring, and confirmation would require future testing under the same conditions.

As a final observation, the high transmission potential found for UC virus in spring Chinook salmon may suggest a unique ecological role that explains, at least in part, the asymmetric prevalence of IHNV subgroups in CRB Chinook salmon. As previously reported, the IHNV detected in CRB Chinook salmon is 83% UC and 17% MD, with this asymmetry found in both spring-and fall-run fish [16]. In our previous work we found that UC and MD viruses did not differ in infectivity for either spring or fall Chinook salmon, so the asymmetry of field occurrence was not due to UC being more infectious [16]. The UC subgroup evolved relatively recently as a generalist lineage infecting all three dominant hosts in the CRB, but it is most prevalent in Chinook salmon [7,8,38]. Here, spring Chinook salmon had uniquely high shedding of UC virus. This suggests that spring Chinook salmon may contribute more than fall-run fish to the dominance of UC in CRB Chinook salmon, despite the greater abundance of fall-run fish [16].

## 5. Conclusions

In North America all IHNV detected to date is in the L, M, or U genogroups, and the great majority of North American IHNV detections are found in one of three host species: Chinook salmon, sockeye salmon, and rainbow/steelhead trout [39]. Here we investigated the shedding of L, UC, and MD virus strains from infected Chinook salmon, and found kinetics very consistent with the acute kinetics described previously for M group strains in rainbow trout [13,14]. In the future it will be interesting to determine if this pattern is also broadly consistent for U, M, and L viruses in all three dominant salmonid hosts, ideally tested with larger numbers of fish and more virus strains within each genogroup. It is important to recognize that the specific timing of pathogen-shedding kinetics can vary with exposure dose and experimental conditions [32,33]. Therefore, comparison of results obtained using the same experimental conditions will be useful for elucidating variation in shedding patterns that are relevant to the ecology of the diverse IHNV subgroups that occur in multi-host ecosystems. We observed here intraspecific variation in which spring-run Chinook salmon shed dramatically more UC subgroups IHNV than fall-run fish. We also found several indications that relative transmission fitness, measured as viral shedding, did not correlate consistently with in-host replication levels or virulence [16]. Overall we found that the host: pathogen interaction of spring Chinook salmon with the generalist UC subgroup IHNV has a highly advantageous combination of high transmission fitness and low virulence that may explain the ecological success of UC IHNV in the CRB [8,38].

## Data Availability

The data are not currently available from the University of Washington. Contact University of Washington for further information.

## Supplementary Materials

The following supporting information can be downloaded at: https://www.mdpi.com/article/10.3390/ani12151887/s1, Table S1: Virus shedding data for six treatment groups of Chinook salmon (spring-run or fall-run) exposed by immersion to IHNV strains from the L, UC, or MD subgroups.
