# Supplementary material for: Shedding Kinetics of Infectious Hematopoietic Necrosis Virus (IHNV) in Juvenile Spring- and Fall-Run Chinook Salmon of the Columbia River Basin
Source: Animals (Basel). 2022 Jul 24;12(15):1887. doi: 10.3390/ani12151887 (PMC9331747; doi:10.3390/ani12151887)
Supplement: Supplementary file 1 [file animals-12-01887-s001.zip › animals-1751428-supplementary.pdf]

Supplemental Table S1. Virus shedding data for six treatment groups of Chinook salmon (spring-run or fall-run) exposed by immersion to IHNV strains from the L, UC, or MD subgroups.

Chinook salmon population	Virus exposure	Number of fish shedding per group	Mean $\log_{10}$ peak quantity[a] of virus shed per fish	Mean $\log_{10}$ total quantity[b] of shed virus per fish	Log_{10} total quantity[c] of virus shed per group
Spring-run	L	9/10	3.65	3.81	4.81
Spring-run	UC	6/10	4.22	4.40	5.84
Spring-run	MD	7/10	3.73	3.90	4.82
Fall-run	L	7/10	3.34	3.46	4.72
Fall-run	UC	1/10	2.88	2.88	2.88
Fall-run	MD	4/10	3.30	3.48	4.37

[a] Mean $\log_{10}$ peak quantity was calculated as the mean of the log transformed peak quantities shed by each fish that shed detectable viral RNA within a treatment group (i.e. only for fish that shed virus).

[b] Mean $\log_{10}$ total quantity was calculated as the mean of the log transformed total quantities shed by each fish over the entire experiment, for fish that shed detectable viral RNA at any time within a treatment group (i.e. only for fish that shed virus).

[c] Log_{10} total quantity of shed virus for each treatment group (including all fish) was calculated as the Log_{10} of the sum of all raw quantities (not log transformed) shed by each fish within each treatment group.
